# A Virtual Community of Practice: An International Educational Series in Pediatric Neurocritical Care

**DOI:** 10.3390/children9071086

**Published:** 2022-07-20

**Authors:** Jennifer C. Erklauer, Ajay X. Thomas, Sue J. Hong, Brian L. Appavu, Jessica L. Carpenter, Nicolas R. Chiriboga-Salazar, Peter A. Ferrazzano, Zachary Goldstein, Jennifer L. Griffith, Kristin P. Guilliams, Matthew P. Kirschen, Karen Lidsky, Marlina E. Lovett, Brandon McLaughlin, Jennifer C. Munoz Pareja, Sarah Murphy, Wendy O’Donnell, James J. Riviello, Michelle E. Schober, Alexis A. Topjian, Mark S. Wainwright, Dennis W. Simon, Pediatric Neurocritical Care Research Group

**Affiliations:** 1 Department of Pediatrics, Division of Pediatric Critical Care Medicine, Texas Children’s Hospital, Baylor College of Medicine, Houston, TX 77030, USA; 2 Department of Pediatrics, Division of Pediatric Neurology and Developmental Neuroscience, Texas Children’s Hospital, Baylor College of Medicine, Houston, TX 77030, USA; ajay.thomas@bcm.edu (A.X.T.); jjriviel@texaschildrens.org (J.J.R.J.); 3 Jan and Dan Duncan Neurological Research Institute, Texas Children’s Hospital, Houston, TX 77030, USA; 4 Department of Pediatrics, Ann & Robert H. Lurie Children’s Hospital of Chicago and Northwestern University Feinberg School of Medicine, Chicago, IL 60611, USA; shong@luriechildrens.org (S.J.H.); nchiribogasalazar@luriechildrens.org (N.R.C.-S.); 5 Division of Neurology, Barrow Neurological Institute at Phoenix Children’s Hospital, University of Arizona College of Medicine, Phoenix, AZ 85004, USA; bappavu@phoenixchildrens.com; 6 Department of Pediatrics, Division of Pediatric Neurology, University of Maryland Children’s Hospital, Baltimore, MD 21201, USA; jessica.carpenter@som.umaryland.edu; 7 Department of Pediatrics, University of Wisconsin, Madison, WI 53706, USA; ferrazzano@pediatrics.wisc.edu; 8 Division of Critical Care, Barrow Neurological Institute at Phoenix Children’s Hospital, University of Arizona College of Medicine, Phoenix, AZ 85004, USA; zgoldstein@phoenixchildrens.com; 9 Department of Neurology, Washington University in St. Louis, St. Louis, MO 63130, USA; griffitj@wustl.edu (J.L.G.); kristinguilliams@wustl.edu (K.P.G.); 10 Department of Pediatrics and Radiology, Washington University in St. Louis, St. Louis, MO 63130, USA; 11 Department of Anesthesiology and Critical Care Medicine, The Children’s Hospital of Philadelphia, School of Medicine, University of Pennsylvania Perelman, Philadelphia, PA 19104, USA; kirschenm@chop.edu (M.P.K.); topjian@chop.edu (A.A.T.); 12 Department of Pediatric Critical Care, Division of Critical Care Medicine, University of Florida Jacksonville and Wolfson Children’s Hospital, Jacksonville, FL 322007, USA; karen.lidsky@jax.ufl.edu; 13 Department of Pediatrics, Division of Critical Care Medicine, Nationwide Children’s Hospital, The Ohio State University, Columbus, OH 43205, USA; marlina.lovett@nationwidechildrens.org; 14 Department of Critical Care Medicine, UPMC Children’s Hospital of Pittsburgh, Pittsburgh, PA 15224, USA; bmclaughlin@pitt.edu (B.M.); wendyodonnell@pitt.edu (W.O.); 15 Department of Pediatric Critical Care Medicine, School of Medicine, University of Miami Miller, Miami, FL 33136, USA; jcm457@med.miami.edu; 16 Department of Pediatrics, Division of Pediatric Critical Care Medicine, Massachusetts General Hospital for Children, Harvard Medical School, Boston, MA 02115, USA; samurphy@mgh.harvard.edu; 17 Department of Pediatrics, Division of Critical Care of the University of Utah, Salt Lake City, UT 84112, USA; michelle.schober@hsc.utah.edu; 18 Division of Neurology, Seattle Children’s Hospital, University of Washington, Seattle, WA 98105, USA; mark.wainwright@seattlechildrens.org; 19 Department of Critical Care Medicine and Pediatrics, UPMC Children’s Hospital of Pittsburgh, Pittsburgh, PA 15224, USA; dennis.simon2@chp.edu

**Keywords:** pediatric neurocritical care, neurocritical care, community of practice, medical education

## Abstract

Pediatric neurocritical care (PNCC) is a rapidly growing field. Challenges posed by the COVID-19 pandemic on trainee exposure to educational opportunities involving direct patient care led to the creative solutions for virtual education supported by guiding organizations such as the Pediatric Neurocritical Care Research Group (PNCRG). Our objective is to describe the creation of an international, peer-reviewed, online PNCC educational series targeting medical trainees and faculty. More than 1600 members of departments such as pediatrics, pediatric critical care, and child neurology hailing from 75 countries across six continents have participated in this series over a 10-month period. We created an online educational channel in PNCC with over 2500 views to date and over 130 followers. This framework could serve as a roadmap for other institutions and specialties seeking to address the ongoing problems of textbook obsolescence relating to the rapid acceleration in knowledge acquisition, as well as those seeking to create new educational content that offers opportunities for an interactive, global audience. Through the creation of a virtual community of practice, we have created an international forum for pediatric healthcare providers to share and learn specialized expertise and best practices to advance global pediatric health.

## 1. Introduction

Pediatric Neurocritical Care (PNCC) has undergone rapid growth as a field since the first PNCC clinical services were formed approximately twenty years ago [1,2,3]. In the United States, multidisciplinary PNCC programs were initially established at five quaternary care pediatric centers [1,2,3,4,5]. Twenty years later, at least 45 U.S. hospitals have an established model of PNCC care by specialized teams [6], and the Neurocritical Care Society now includes a pediatric section, numbering more than 70 active members, as well as pediatric representatives to the majority of its committees and governing bodies. The establishment of multi-center research collaboration, evidence-based treatment guidelines, and training programs has propelled the movement for specialization in PNCC. The need to support educational initiatives in PNCC is evident. To date, however, a clear standard for PNCC education is lacking. Efforts are underway to address the dearth of formal training program requirements and unified curriculum amongst programs [7,8,9].

The goal of advocating for a more standardized curriculum across PNCC training programs coincided with changes in medical education delivery prompted by the COVID-19 pandemic [10,11]. The disruptions imposed by the pandemic challenged the educators and program developers to devise innovative approaches to deliver quality medical education. Many healthcare systems limited student and trainee contact with patients to minimize possible viral exposure, and in pediatrics, inpatient hospitalization volumes plummeted [12]. To address vast concerns that limiting necessary patient care experience and decreased patient volumes could compromise learner competency, programs were tasked to rapidly pivot existing educational activities to online platforms and create unique educational experiences to supplement patient care encounters [13].

Concurrent with these initiatives, the Pediatric Neurocritical Care Research Group (PNCRG), a backbone organization within PNCC, expanded its mission to include education and advocacy. The PNCRG held several sessions to deliberate the best ways to advance its mission to not only benefit its members but also to address the needs of global PNCC providers. It was determined that developing opportunities for the sharing of expertise and management approaches from an extended international community would be important.

Globally, support for PNCC has been increasing. Recent prevalence studies indicate that pediatric critical neurologic illnesses is common worldwide [14]. Health systems across the world are responding by evaluating current clinical programs for patients with critical neurologic illness and discussing goals for the future [15,16,17,18,19,20,21]. International societies are working on expanding neurocritical care educational initiatives and clinician expertise with focused programs at annual meetings, and individual healthcare systems are offering clinical courses and workshops. It has been challenging, however, for the growing international PNCC community to engage together in ongoing, consistent collaborative educational efforts. There has been no clear virtual forum to disseminate expertise more broadly in PNCC or to discuss clinical management approaches across institutions and countries.

The following describes the process undertaken to develop an online, peer-reviewed, collaborative, multi-disciplinary, international educational series in PNCC, which has encouraged the development of an expansive virtual PNCC community of practice. This framework could serve as a guide for other organizations to create educational curricula in PNCC as well as other medical specialties.

## 2. Materials and Methods

### 2.1. Context

The PNCRG, a guiding organization in PNCC with an international membership, represents the global *Community of Practice* (CoP) of PNCC. It has been the primary forum where clinicians and researchers interested in PNCC from multiple institutions work collaboratively to advance the field consistent with the three tenants of the CoP; *domain*, *community*, and *practice* [22]. In this model of social learning, the domain is the shared interest in the field of PNCC, the community is the group of multi-disciplinary clinicians and researchers focused on collaboration and knowledge sharing for the advancement of the field of PNCC, and the practice is the development of optimal clinical applications in PNCC through knowledge sharing, research, and collaboration. This ongoing CoP within the PNCRG allowed for the rapid development of a virtual PNCC community of practice as well as its accelerated expansion for global outreach during the COVID-19 pandemic.

### 2.2. Program Description

Acknowledging that many institutions were already developing the PNCC lecture series, a cooperative expansion of the individual series was proposed to the PNCC fellowship program directors and the leadership of the PNCRG to transpire collective impact. We convened a planning committee of faculty and trainees in pediatric neurology, pediatric critical care, and PNCC from multiple institutions as well as a medical education specialist to determine the needs, the content, the instructional processes, and the evaluation of this collaborative educational series. The committee derived consensus on (1) goals for the series, (2) target audience, (3) format for the series, (4) essential topics, and (5) expert speakers to lead each session.

1. Goal: The series aimed to create a peer-reviewed online educational series with an advanced discussion of core PNCC principles led by experts in the field.
2. Target audience: The series was designed to deliver content relevant to trainees in PNCC directly. We encouraged PNCC faculty to attend by including expert-level discussions of interest to both trainees and faculty. To further interprofessional education at all levels, the series was open to faculty, fellow and resident trainees, medical students, advanced practice providers, nurses, pharmacists, and therapists.
3. Format: The monthly webinar series employed multimodality instructional approaches to deliver case-based discussion for knowledge content updates and high-order critical thinking. Each topic was structured to be delivered over two distinct, consecutive sessions. The first session was a didactic lecture delivered by a content expert, providing an up-to-date overview of the neurocritical care topic, including the most important landmark articles to provide a strong foundation. This was followed by a second session, an in-depth expert panel discussion of challenging clinical cases for that topic, led by the previous session’s speaker. The educational series used audience polling questions to optimize the engagement of participants and launch expert discussion.
4. Essential topics: The expert group consensus meetings derived high yield topics and categorized them in order of importance to prioritize the chronologic delivery of content and to adapt in real-time to the needs of the field.
5. Expert speakers: Lead speakers for each session were identified through group consensus by the planning committee based on their contributions to the literature or participation in the development of PNCC clinical guidelines. Along with the planning committee, the lead speakers selected members to serve as the panelists for the subsequent case-based discussion, delineate learning objectives, and identify clinical cases.

For peer review, we used a subcommittee comprised of faculty and trainees in pediatric critical care, pediatric neurology, and PNCC. This group reviewed the planned presentation and provided feedback to speakers to ensure that standards for high-quality content were met. Questions for peer review were employed (Box 1).

We used Zoom (Zoom Video Communications, Inc., San Jose, CA, USA) as the main online platform for participation through The University of Pittsburgh Department of Critical Care Medicine, which also provided support for registration, video editing, and advertising of the sessions. Each lecture was recorded and uploaded to a ‘Pediatric Neurocritical Care’ channel on the platform Vimeo (Vimeo Inc., New York, NY, USA) (https://vimeo.com/channels/pncc accessed on 20 May 2022). The recorded sessions have remained available for viewing online with free access.

We leveraged the existing infrastructure to advertise the activities (Twitter, PNCRG member list serve, and fellowship director list serves). Advertisements included the registration link for access to the series.

A series email was provided (PNCC.Lectures@gmail.com) to aid in direct communication between the audience and series/session leaders. Participants were encouraged to email questions, feedback, and topic/case recommendations for future sessions. When appropriate, questions after the didactic sessions were incorporated into the case-based discussions the following month.

The series was accredited for continuing medical education through the Texas Children’s Hospital and their existing PNCC educational series. Session leaders, panelists, and the planning committee were required to declare any conflict of interest. Participation in the educational series was free. Evaluation forms (outlined in Box 2) were disseminated to all participants following the live sessions.

Box 1 **Questions for peer review.**

1. Is this lecture free from commercial bias?

2. Are conflicts of interest disclosed?

3. Does this lecture content support neurocritical care trainees by delivering necessary foundational knowledge on this subject?

4. Does the lecture content meet the typical neurocritical care trainee at their level of training?

5. Does this lecture content include evidence-based guidance where available?

6. Does this lecture only take one point of view on diagnosis/management when other approaches may also be reasonable? If yes, does the speaker convey this?

7. Do you have any additional feedback for the lecturer?

Box 2 **Evaluation for educational session.**

For questions 1–7, answer choices are Strongly Disagree, Disagree, Neutral, Agree, and Strongly Agree.

1. The speaker was well prepared for the session and spoke clearly and audibly.
2. The objectives were clearly delineated.
3. The content of the session was appropriate and understandable.
4. The session emphasized new concepts.
5. The information presented will be useful in my field.
6. The session improved my knowledge of this topic.
7. The goals and objectives of the session were met.

For questions 8–10, participants are asked to rate from 1 (not at all) to 5 (very) both before and after the session.

8. How familiar are you regarding (the topic)?
9. How confident are you in describing (the topic/learning objectives for the topic)?
10. How familiar are you regarding (the topic/learning objectives for the topic)?

For questions 11 and 13, answer choices are Yes/No.

11. Do you intend to make changes as a result of today’s activity?
12. If yes, list at least one change you intend to make.
13. Did you notice commercial bias in this session?

For question 14, answer choices are Below Expectations, Meets Expectations, and Exceeds Expectations.

14. Rate the overall quality of the session.
  Question 15 is a free text answer.

15. How could this session be more productive?

## 3. Results

**Figure 1:**
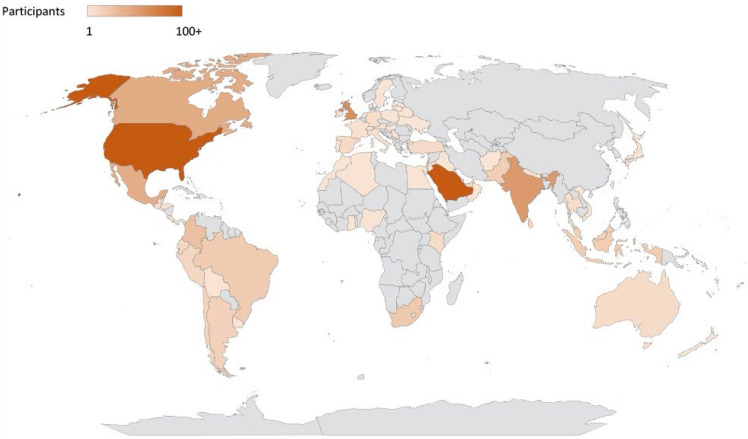
Participants in the PNCC Educational Series.

During the first 10 months of the series, five core topics in PNCC were covered (Table 1) over 10 sessions. Session leaders and moderators represented institutions in Canada and the United States. Expert panelists for the sessions represented institutions in Canada, Colombia, the United Kingdom, and the United States. A total of 1616 unique participants from 75 countries across six continents registered for at least one of the sessions (Figure 1). To date, 33% of registrants reported hearing about the series through social media, 30% through PNCRG emails, 16% through word of mouth, and 11% through their Residency/Fellowship Program Director.

Participants represented a range of health professionals and training levels (Table 2). Although our target audience was trainees, attending physicians accounted for 48% of the participants. Another 8% of the registrants identified their position as ‘Staff,’ many of whom were faculty physicians. Physician trainees at the resident and fellow level accounted for 34% of the participants, with an additional 1% participation from medical students. Ninety-seven (6%) of the participants were advanced practice providers, including nurse practitioners and physician assistants, and fifty-three (3%) were registered nurses. The most common appointments were held in the departments of critical care medicine, pediatrics, and neurology. Many of the participants held appointments in multiple departments (Table 3).

The online channel for ‘Pediatric Neurocritical Care’ had 10 uploaded videos of the recorded live sessions at https://vimeo.com/channels/pncc (accessed on 20 May 2022). There were 2551 views and 131 followers.

**Figure 2:**
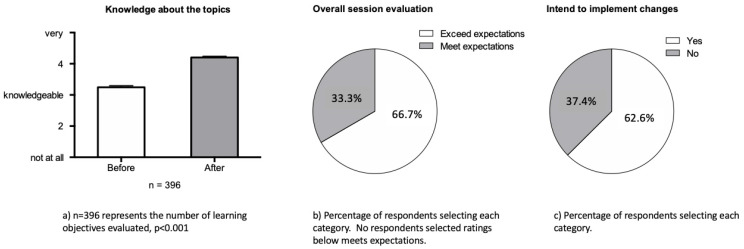
Attendee evaluation of the educational series. a) Knowledge about the topics. *n* = 396 represents the number of learning objectives evaluated, p <0.001. b) Overall session evaluation. Percentage of respondents selecting each category. No respondents selected ratings below meets expectations. c) Intend to implement changes. Percentage of respondents selecting each category.

Through the evaluation process, participants reported improved knowledge after attending the educational sessions overall. The majority of the evaluation respondents reported that the educational session exceeded their expectations, and they intended to implement changes to their practices as a result of what they had learned (Figure 2).

## 4. Discussion

We created a virtual community of practice with international, interprofessional, and multi-institutional collaboration for advanced topics in medical education, and participants reported improved knowledge with many planning to implement practice changes as a result. The concept of community of practice has been applied in many industries, including healthcare, as a framework for collaborative social learning [22,23,24]. The steps used to create and advance our PNCC Educational Series in conjunction with the PNCRG allowed us to create a forum comprised of an international team with a shared interest and common identity in PNCC with the goal of fostering knowledge sharing of best care practices for children with critical neurologic illness. While a community of practice in PNCC has existed for many years in organizations such as the PNCRG, the development of a virtual community of practice with an expanded global reach was new. By utilizing existing networks combined with the expansion of innovative technology and online learning models, we have been able to partner with clinicians across the globe to enhance the educational experience. 

While many institutions in the United States have created a model for PNCC care (35%), many institutions in the U.S. and throughout the world do not have access to this type of sub-specialty care [6]. Some of the literature on patients treated in centers with dedicated PNCC programs suggest improved outcomes, thereby creating a possible disparity in the care provided to children with critical neurologic illness [25]. For some conditions, such as acute ischemic stroke, management guidelines recommend establishing networks with tertiary centers with expertise in neurocritical care and other specialized groups for those caring for children with acute ischemic stroke [26]. Having access to a global community of practice such as ours can help clinicians provide quality patient care to these patients without immediate access to specialists in PNCC. 

Even within large institutions with established PNCC programs, there may be only a few experts in PNCC at each location. While there is growing research evidence to assist in the creation of evidence-based protocols and guidelines, many controversial topics and aspects of care remain unresolved. Bringing together multi-institutional interprofessional experts with various viewpoints induces expansive learning where nuances of the care for these critically ill children can be discussed, and innovative ideas and collective wisdom can manifest. 

Participating in a real-time, interactive discussion with leaders in the field addressing best practices for real-life scenarios is invaluable. This is evidenced by the number of faculty who participated in the series despite the direct target audience being trainees and who reported intentions to change practice. This may be indicative of faculty and trainees encountering similar challenges caring for patients at the bedside or may suggest that formal training programs in pediatric critical care and pediatric neurology could benefit from enhanced PNCC educational programs. While efforts are ongoing to standardize formal training requirement goals and recommendations in PNCC, we look for this educational series to supplement these initiatives to ensure we continue to meet the needs of those providing care in the field of PNCC. 

Insufficient resources, such as dedicated time and funding support, pose potential obstacles in developing a series such as this one. To date, we have relied primarily on volunteers from the PNCRG, such as members of the PNCRG Educational Committee and PNCC fellowship directors, to help accomplish the necessary work. Partnering with the guiding organizations in each field, such as PNCRG in PNCC, was important to gain momentum for the project. We obtained support from the home institutions of the leadership group for the series, such as CME availability, technological support, and additional resources. Finding organizations whose goals align with the aims of the educational initiative and who are willing to contribute to the critical mission was important. As the series expands, dedicated funding in the form of educational grants will likely become necessary to continue to advance the series. 

As a result of feedback from participants in the series and the planning committee, we look to continue to grow, refine, and enhance this series over time. We are working to increase the frequency of the sessions with the inclusion of additional formats to highlight procedural skills and critical appraisal of the PNCC literature. We look to augment educational goals with the creation of entrusted professional activities, and we aim to expand our global reach through the translation of the sessions into additional languages and dissemination through outreach organizations. 

While the COVID-19 pandemic appears to be waning, it has forever transformed our world, promoting online learning, communication, and collaboration that is likely to stay. As such, our educational series can serve as a guide to others looking to strengthen partnerships for educational initiatives across the globe. 

## 5. Conclusions

We have shown that it is possible to create a multi-institutional, interprofessional, peer-reviewed international pediatric educational series with experts in the field. In a relatively short period of time, we demonstrated significant global participation with high rates of attendance and reported knowledge uptake and intent to change practice. This experience serves as a guide for those looking to expand global educational outreach by expanding and building a virtual community of practice. This series has the potential to impact knowledge across the world, facilitating our ability to learn from each other.

## Figures and Tables

**Table 1:** Initial core topics as decided by expert consensus.

Topic	Leader
Cardiopulmonary Resuscitation and Post-Cardiac Arrest Care in Children	Alexis Topjian Matthew Kirschen
Fundamentals of Acute Ischemic Stroke in Children	Kristin Guilliams
Refractory Status Epilepticus in Children	James J. Riviello
Neurologic Manifestations of COVID-19 in Children in the Pediatric Intensive Care Unit	Michelle Schober Rebecca Riggs
Acute Spinal Cord Injury	Michael Fehlings

**Table 2:** Self-reported position of participants in the Pediatric Neurocritical Care Education Series.

Position	Participants *n* = 1616 (%)
Attending	776 (48)
Fellow	350 (22)
Resident	198 (12)
Staff	128 (8)
Advanced Practice Provider	97 (6)
Registered nurse	53 (3)
Medical Student	14 (1)

**Table 3:** Self-reported department(s) affiliations of participants in the Pediatric Neurocritical Care Education Series.

Department	Participants *n* = 1616 (%)
Critical Care Medicine	824 (51)
Pediatrics	799 (49)
Neurology	286 (18)
Neurocritical Care	80 (5)
Emergency Medicine	40 (2)
Anesthesiology	35 (2)
Physical Medicine and Rehabilitation	22 (1)
Neurosurgery	16 (1)

The total exceeds 100% due to multiple appointments by some participants.

## Data Availability

Not applicable.

## References

[1] Bell M.J., Carpenter J., Au A.K., Keating R.F., Myseros J.S., Yaun A., Weinstein S. (2009). Development of a Pediatric Neurocritical Care Service. Neurocrit. Care.

[2] Tasker R.C. (2009). Pediatric Neurocritical Care: Is It Time to Come of Age?. Curr. Opin. Pediatr..

[3] Wainwright M.S., Hansen G., Piantino J. (2016). Pediatric Neurocritical Care in the 21st Century: From Empiricism to Evidence. Curr. Opin. Crit. Care.

[4] Horvat C.M., Mtaweh H., Bell M.J. (2016). Management of the Pediatric Neurocritical Care Patient. Semin. Neurol..

[5] LaRovere K.L., Riviello J.J. (2008). Emerging Subspecialties in Neurology: Building a Career and a Field: Pediatric Neurocritical Care. Neurology.

[6] LaRovere K.L., Murphy S.A., Horak R., Vittner P., Kapur K., Proctor M., Tasker R.C. (2018). Pediatric Neurocritical Care: Evolution of a New Clinical Service in PICUs across the United States. Pediatr. Crit. Care Med..

[7] Lee J.C., Riviello J.J. (2011). Education of the Child Neurologist: Pediatric Neurocritical Care. Semin. Pediatr. Neurol..

[8] Scher M. (2008). Proposed Cross-Disciplinary Training in Pediatric Neurointensive Care. Pediatr. Neurol..

[9] LaRovere K.L., Graham R.J., Tasker R.C. (2013). Pediatric Critical Nervous System Program (pCNSp) Pediatric Neurocritical Care: A Neurology Consultation Model and Implication for Education and Training. Pediatr. Neurol..

[10] Papapanou M., Routsi E., Tsamakis K., Fotis L., Marinos G., Lidoriki I., Karamanou M., Papaioannou T.G., Tsiptsios D., Smyrnis N. (2022). Medical Education Challenges and Innovations during COVID-19 Pandemic. Postgrad. Med. J..

[11] Woolliscroft J.O. (2020). Innovation in Response to the COVID-19 Pandemic Crisis. Acad. Med..

[12] Pelletier J.H., Rakkar J., Au A.K., Fuhrman D., Clark R.S.B., Horvat C.M. (2021). Trends in US Pediatric Hospital Admissions in 2020 Compared with the Decade before the COVID-19 Pandemic. JAMA Netw. Open.

[13] Connolly N., Abdalla M.E. (2022). Impact of COVID-19 on Medical Education in Different Income Countries: A Scoping Review of the Literature. Med. Educ. Online.

[14] Fink E.L., Kochanek P.M., Tasker R.C., Beca J., Bell M.J., Clark R.S.B., Hutchison J., Vavilala M.S., Fabio A., Angus D.C. (2017). International Survey of Critically Ill Children with Acute Neurologic Insults: The Prevalence of Acute Critical Neurological Disease in Children: A Global Epidemiological Assessment Study. Pediatr. Crit. Care Med..

[15] Fink E.L., von Saint Andre-von Arnim A., Kumar R., Wilson P.T., Bacha T., Aklilu A.T., Teklemariam T.L., Hooli S., Tuyisenge L., Otupiri E. (2018). Traumatic Brain Injury and Infectious Encephalopathy in Children from Four Resource-Limited Settings in Africa. Pediatr. Crit. Care Med..

[16] Wang H., Zhao S., Wang S., Zheng Y., Wang S., Chen H., Pang J., Ma J., Yang X., Chen Y. (2022). Global Magnitude of Encephalitis Burden and Its Evolving Pattern over the Past 30 Years. J. Infect..

[17] Akinyemi R.O., Ovbiagele B., Adeniji O.A., Sarfo F.S., Abd-Allah F., Adoukonou T., Ogah O.S., Naidoo P., Damasceno A., Walker R.W. (2021). Stroke in Africa: Profile, Progress, Prospects and Priorities. Nat. Rev. Neurol..

[18] Shlobin N.A., Radwanski R.E., Sandhu M.R.S., Rosseau G., Dahdaleh N.S. (2022). Increasing Equity in Medical Student Neurosurgery Education through Distance Learning. World Neurosurg..

[19] Du R.Y., Thiong’o G.M., LoPresti M.A., Mohan N.K., Dewan M.C., Lepard J., Lam S. (2020). Pediatric Neurosurgery in East Africa: An Education and Needs-Based Survey. World Neurosurg..

[20] Spanu F., Piquer J., Panciani P.P., Qureshi M.M. (2018). Practical Challenges and Perspectives for the Development of Neurosurgery in a Peripheral East African Hospital during a One-Volunteer Midterm Mission. World Neurosurg..

[21] Brown M.W., Foy K.E., Chanda C., Mulundika J., Koralnik I.J., Siddiqi O.K. (2018). Neurologic Illness in Zambia: A Neurointensivist’s Experience. J. Neurol. Sci..

[22] Wenger E. (1998). Communities of Practice: Learning, Meaning, and Identity; Learning in Doing.

[23] Cruess R.L., Cruess S.R., Steinert Y. (2018). Medicine as a Community of Practice: Implications for Medical Education. Acad. Med..

[24] O’Brien B.C., Battista A. (2020). Situated Learning Theory in Health Professions Education Research: A Scoping Review. Adv. Health Sci. Educ. Theory Pract..

[25] Pineda J.A., Leonard J.R., Mazotas I.G., Noetzel M., Limbrick D.D., Keller M.S., Gill J., Doctor A. (2013). Effect of Implementation of a Paediatric Neurocritical Care Programme on Outcomes after Severe Traumatic Brain Injury: A Retrospective Cohort Study. Lancet Neurol..

[26] Ferriero D.M., Fullerton H.J., Bernard T.J., Billinghurst L., Daniels S.R., DeBaun M.R., deVeber G., Ichord R.N., Jordan L.C., Massicotte P. (2019). Management of Stroke in Neonates and Children: A Scientific Statement from the American Heart Association/American Stroke Association. Stroke.

